# 3-(2-Bromo­phen­yl)thia­zolo[3,2-*a*]benzimidazole

**DOI:** 10.1107/S1600536811034842

**Published:** 2011-08-31

**Authors:** Zhi-Ming Wang, Bin Yu, Yuan Cui, Xiu-Qing Zhang, Xiao-Qiang Sun

**Affiliations:** a School of Petrochemical Engineering, Changzhou University, Changzhou 213164, People’s Republic of China; b High Technology Research Institute of Nanjing University, Changzhou 213162, People’s Republic of China

## Abstract

The title compound, C_15_H_9_BrN_2_S, was prepared by the reaction of 1-bromo-2-(2,2-dibromo­vin­yl)benzene with 1*H*-benzo[*d*]imidazole-2(3*H*)-thione. The thia­zolo[3,2-*a*]benz­imidazole fused-ring system is nearly planar, the maximum atomic deviation being 0.049 (4) Å. This mean plane is oriented at a dihedral angle of 71.55 (17)° with respect ot the bromo­phenyl ring. π–π stacking is observed in the crystal structure, the centroid–centroid distance between the thia­zole and imidazole rings of adjacent mol­ecules being 3.582 (2) Å.

## Related literature

For the biological activity of imidazoles and their use as inhibitors of neurodegenerative disorders and as anti­tumor drugs, see: Park *et al.* (1977 ▶); Schuckmann *et al.* (1979 ▶). For related imidazole compounds, see: Andreani *et al.* (2005 ▶); Xu *et al.* (2010 ▶).
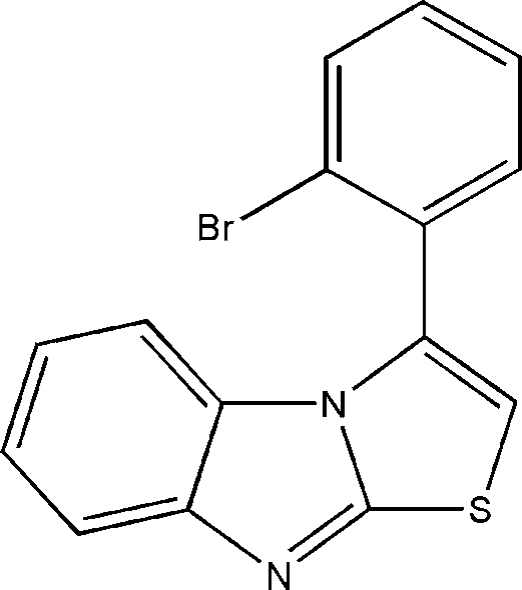


## Experimental

### 

#### Crystal data

C_15_H_9_BrN_2_S
*M* *_r_* = 329.21
Monoclinic, 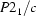

*a* = 11.2459 (19) Å
*b* = 9.1554 (16) Å
*c* = 14.2842 (18) Å
β = 118.159 (9)°
*V* = 1296.6 (4) Å^3^
*Z* = 4
Mo *K*α radiation
μ = 3.32 mm^−1^
*T* = 296 K
0.24 × 0.22 × 0.22 mm

#### Data collection

Bruker APEXII CCD diffractometer
Absorption correction: multi-scan (*SADABS*; Bruker, 2006 ▶) *T*_min_ = 0.456, *T*_max_ = 0.483
7492 measured reflections
2533 independent reflections
1977 reflections with *I* > 2σ(*I*)
*R*_int_ = 0.073

#### Refinement

*R*[*F* ^2^ > 2σ(*F* ^2^)] = 0.047
*wR*(*F* ^2^) = 0.120
*S* = 1.01
2533 reflections
173 parameters
H-atom parameters constrained
Δρ_max_ = 0.52 e Å^−3^
Δρ_min_ = −1.20 e Å^−3^

### 

Data collection: *APEX2* (Bruker, 2006 ▶); cell refinement: *SAINT* (Bruker, 2006 ▶); data reduction: *SAINT*; program(s) used to solve structure: *SHELXTL* (Sheldrick, 2008 ▶); program(s) used to refine structure: *SHELXTL*; molecular graphics: *SHELXTL*; software used to prepare material for publication: *SHELXTL*.

## Supplementary Material

Crystal structure: contains datablock(s) I, global. DOI: 10.1107/S1600536811034842/xu5299sup1.cif

Structure factors: contains datablock(s) I. DOI: 10.1107/S1600536811034842/xu5299Isup2.hkl

Supplementary material file. DOI: 10.1107/S1600536811034842/xu5299Isup3.cml

Additional supplementary materials:  crystallographic information; 3D view; checkCIF report

## Figures and Tables

**Fig. 1.**
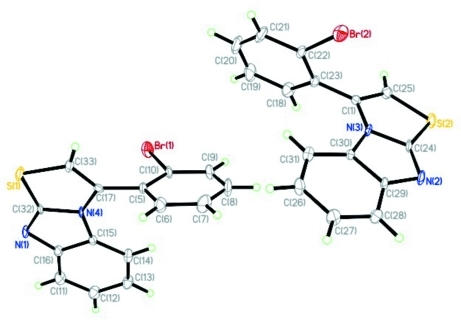
The molecular structure.

## supplementary crystallographic information

### Comment

Owing to the promising biological activities as inhibitors of neurodegenerative disorders and antitumor drugs, such compound structures have been studied (Park *et al.*, 1977; Schuckmann *et al.*, 1979). In the past decades, most of these investigations were carried out with imidazole (Andreani *et al.*, 2005; Xu *et al.*, 2010). We herein present the structure of 3-(2-bromopheny)thiazolo[3,2-*a*]benzimidazole(Fig. 1).

In the title compound, the benzene imidazole ring and thiazole ring are almost in the same plane. In the crystal structure, π-π interactions contribute the crystal packing.

### Experimental

1-Bromo-2-(2,2-dibromovinyl)benzene(1.2 mmol) in 1.0 ml of DMF were added to a stirred solution of 1*H*-benzo[*d*]imidazole-2(3*H*)-thione(1.0 mmol), Cs_2_CO_3_(2 mmol), CuI(0.1 mmol) and dmeda(0.2 mmol) in DMF(3 ml) under nitrogen. The resulting mixture was stirred at 100 °C for 4 h. After being cooled to room temperature, the reaction mixture was diluted with water and extracted with CHCl_3,_ the combined organic layer were dried over Na_2_SO_4_ and concentrated. The crude product was further purified by flash column chromatography using petroleum ether (PE) and CH_2_Cl_2_ as a white solid (90% yield). Single crystals suitable for X-ray diffraction were obtained by evaporation of an ethanol solution.

### Refinement

All the H atoms were placed in geometrically idealized positions and constrained to ride on their parent atoms, with C—H distances of 0.93 Å, and with *U*_iso_(H)= 1.2 *U*_eq_(C).

### Crystal data

**Table:**

C_15_H_9_BrN_2_S	*F*(000) = 656
*M**_r_* = 329.21	*D*_x_ = 1.686 Mg m^−^^3^
Monoclinic, *P*2_1_/*c*	Mo *K*α radiation, λ = 0.71073 Å
Hall symbol: -P 2ybc	Cell parameters from 3173 reflections
*a* = 11.2459 (19) Å	θ = 2.8–27.3°
*b* = 9.1554 (16) Å	µ = 3.32 mm^−^^1^
*c* = 14.2842 (18) Å	*T* = 296 K
β = 118.159 (9)°	Block, colourless
*V* = 1296.6 (4) Å^3^	0.24 × 0.22 × 0.22 mm
*Z* = 4	

### Data collection

**Table:**

Bruker APEXII CCD diffractometer	2533 independent reflections
Radiation source: fine-focus sealed tube	1977 reflections with *I* > 2σ(*I*)
graphite	*R*_int_ = 0.073
φ and ω scans	θ_max_ = 26.0°, θ_min_ = 2.1°
Absorption correction: multi-scan (*SADABS*; Bruker, 2006)	*h* = −13→11
*T*_min_ = 0.456, *T*_max_ = 0.483	*k* = −11→10
7492 measured reflections	*l* = −11→17

### Refinement

**Table:**

Refinement on *F*^2^	Secondary atom site location: difference Fourier map
Least-squares matrix: full	Hydrogen site location: inferred from neighbouring sites
*R*[*F*^2^ > 2σ(*F*^2^)] = 0.047	H-atom parameters constrained
*wR*(*F*^2^) = 0.120	*w* = 1/[σ^2^(*F*_o_^2^) + (0.0717*P*)^2^] where *P* = (*F*_o_^2^ + 2*F*_c_^2^)/3
*S* = 1.01	(Δ/σ)_max_ = 0.001
2533 reflections	Δρ_max_ = 0.52 e Å^−^^3^
173 parameters	Δρ_min_ = −1.20 e Å^−^^3^
0 restraints	Extinction correction: *SHELXTL* (Sheldrick, 2008), Fc^*^=kFc[1+0.001xFc^2^λ^3^/sin(2θ)]^-1/4^
Primary atom site location: structure-invariant direct methods	Extinction coefficient: 0.198 (7)

### Special details

**Table:**

Geometry. All e.s.d.'s (except the e.s.d. in the dihedral angle between two l.s. planes) are estimated using the full covariance matrix. The cell e.s.d.'s are taken into account individually in the estimation of e.s.d.'s in distances, angles and torsion angles; correlations between e.s.d.'s in cell parameters are only used when they are defined by crystal symmetry. An approximate (isotropic) treatment of cell e.s.d.'s is used for estimating e.s.d.'s involving l.s. planes.
Refinement. Refinement of *F*^2^ against ALL reflections. The weighted *R*-factor *wR* and goodness of fit *S* are based on *F*^2^, conventional *R*-factors *R* are based on *F*, with *F* set to zero for negative *F*^2^. The threshold expression of *F*^2^ > σ(*F*^2^) is used only for calculating *R*-factors(gt) *etc*. and is not relevant to the choice of reflections for refinement. *R*-factors based on *F*^2^ are statistically about twice as large as those based on *F*, and *R*- factors based on ALL data will be even larger.

### Fractional atomic coordinates and isotropic or equivalent isotropic displacement parameters (Å^2^)

**Table:**

	*x*	*y*	*z*	*U*_iso_*/*U*_eq_	
Br1	0.22834 (4)	0.45478 (5)	0.55293 (3)	0.0445 (2)	
C1	0.2162 (3)	0.0447 (4)	0.5360 (3)	0.0337 (9)	
H1	0.1460	0.0949	0.4811	0.040*	
C2	0.1918 (4)	−0.0647 (4)	0.5901 (3)	0.0414 (10)	
H2	0.1033	−0.0901	0.5711	0.050*	
C3	0.2968 (4)	−0.1382 (4)	0.6728 (3)	0.0407 (9)	
H3	0.2766	−0.2115	0.7082	0.049*	
C4	0.4298 (4)	−0.1059 (4)	0.7041 (3)	0.0389 (9)	
H4	0.4991	−0.1562	0.7596	0.047*	
C5	0.4574 (3)	0.0052 (4)	0.6498 (3)	0.0295 (8)	
C6	0.3489 (3)	0.0771 (3)	0.5661 (3)	0.0239 (7)	
C7	0.5470 (3)	0.1543 (4)	0.5890 (3)	0.0271 (7)	
C8	0.4831 (3)	0.3354 (4)	0.4467 (3)	0.0315 (8)	
H8	0.4781	0.4050	0.3975	0.038*	
C9	0.3737 (3)	0.2780 (4)	0.4475 (3)	0.0258 (7)	
C10	0.2316 (3)	0.3134 (4)	0.3760 (3)	0.0282 (7)	
C11	0.1737 (4)	0.2726 (5)	0.2701 (3)	0.0429 (9)	
H11	0.2244	0.2219	0.2449	0.052*	
C12	0.0396 (5)	0.3077 (5)	0.2014 (4)	0.0592 (13)	
H12	0.0014	0.2812	0.1302	0.071*	
C13	−0.0352 (4)	0.3798 (6)	0.2377 (4)	0.0589 (13)	
H13	−0.1247	0.4020	0.1912	0.071*	
C14	0.0187 (4)	0.4208 (5)	0.3418 (4)	0.0474 (11)	
H14	−0.0338	0.4697	0.3662	0.057*	
C15	0.1524 (3)	0.3888 (4)	0.4105 (3)	0.0297 (8)	
N1	0.4104 (2)	0.1755 (3)	0.5283 (2)	0.0250 (6)	
N2	0.5811 (3)	0.0540 (3)	0.6619 (2)	0.0322 (7)	
S1	0.63442 (8)	0.26789 (10)	0.54574 (8)	0.0354 (3)	

### Atomic displacement parameters (Å^2^)

**Table:**

	*U*^11^	*U*^22^	*U*^33^	*U*^12^	*U*^13^	*U*^23^
Br1	0.0387 (3)	0.0568 (3)	0.0447 (3)	0.00761 (17)	0.0251 (2)	−0.00393 (19)
C1	0.0233 (18)	0.041 (2)	0.031 (2)	0.0051 (15)	0.0084 (16)	0.0037 (16)
C2	0.031 (2)	0.053 (2)	0.043 (2)	−0.0045 (18)	0.0195 (19)	0.0010 (19)
C3	0.039 (2)	0.047 (2)	0.029 (2)	−0.0060 (18)	0.0110 (18)	0.0045 (18)
C4	0.038 (2)	0.042 (2)	0.0250 (19)	0.0043 (18)	0.0055 (17)	0.0051 (17)
C5	0.0222 (17)	0.0344 (17)	0.0216 (17)	0.0006 (15)	0.0019 (15)	−0.0037 (15)
C6	0.0208 (16)	0.0267 (16)	0.0207 (16)	0.0022 (13)	0.0068 (14)	−0.0020 (13)
C7	0.0158 (15)	0.0326 (17)	0.0258 (18)	0.0004 (14)	0.0040 (14)	−0.0081 (15)
C8	0.0235 (17)	0.0360 (19)	0.034 (2)	−0.0002 (15)	0.0129 (16)	−0.0026 (16)
C9	0.0224 (16)	0.0286 (17)	0.0263 (17)	0.0033 (13)	0.0114 (15)	−0.0006 (14)
C10	0.0226 (16)	0.0301 (17)	0.0272 (18)	0.0010 (14)	0.0078 (15)	0.0053 (15)
C11	0.040 (2)	0.049 (2)	0.031 (2)	0.0004 (18)	0.0089 (19)	−0.0024 (18)
C12	0.043 (3)	0.068 (3)	0.035 (2)	−0.001 (2)	−0.008 (2)	0.004 (2)
C13	0.025 (2)	0.067 (3)	0.057 (3)	0.002 (2)	−0.003 (2)	0.012 (3)
C14	0.025 (2)	0.051 (2)	0.063 (3)	0.0114 (18)	0.018 (2)	0.014 (2)
C15	0.0177 (16)	0.0358 (19)	0.0328 (19)	0.0018 (14)	0.0098 (15)	0.0038 (15)
N1	0.0135 (13)	0.0307 (14)	0.0253 (15)	0.0040 (11)	0.0046 (12)	−0.0023 (12)
N2	0.0181 (14)	0.0375 (16)	0.0261 (16)	0.0029 (12)	−0.0017 (13)	−0.0020 (14)
S1	0.0167 (4)	0.0451 (6)	0.0409 (6)	−0.0022 (4)	0.0107 (4)	−0.0042 (4)

### Geometric parameters (Å, °)

**Table:**

Br1—C15	1.895 (4)	C8—C9	1.343 (5)
C1—C2	1.370 (5)	C8—S1	1.735 (4)
C1—C6	1.377 (5)	C8—H8	0.9300
C1—H1	0.9300	C9—N1	1.390 (4)
C2—C3	1.388 (6)	C9—C10	1.470 (5)
C2—H2	0.9300	C10—C11	1.386 (5)
C3—C4	1.376 (6)	C10—C15	1.388 (5)
C3—H3	0.9300	C11—C12	1.395 (6)
C4—C5	1.400 (6)	C11—H11	0.9300
C4—H4	0.9300	C12—C13	1.351 (7)
C5—N2	1.392 (5)	C12—H12	0.9300
C5—C6	1.405 (5)	C13—C14	1.367 (7)
C6—N1	1.391 (4)	C13—H13	0.9300
C7—N2	1.304 (5)	C14—C15	1.385 (5)
C7—N1	1.375 (4)	C14—H14	0.9300
C7—S1	1.732 (4)		
			
C2—C1—C6	117.2 (3)	C8—C9—C10	127.4 (3)
C2—C1—H1	121.4	N1—C9—C10	121.7 (3)
C6—C1—H1	121.4	C11—C10—C15	118.2 (3)
C1—C2—C3	121.3 (4)	C11—C10—C9	119.6 (3)
C1—C2—H2	119.4	C15—C10—C9	122.2 (3)
C3—C2—H2	119.4	C10—C11—C12	120.0 (4)
C4—C3—C2	122.0 (4)	C10—C11—H11	120.0
C4—C3—H3	119.0	C12—C11—H11	120.0
C2—C3—H3	119.0	C13—C12—C11	120.3 (4)
C3—C4—C5	117.9 (3)	C13—C12—H12	119.8
C3—C4—H4	121.1	C11—C12—H12	119.8
C5—C4—H4	121.1	C12—C13—C14	121.0 (4)
N2—C5—C4	129.4 (3)	C12—C13—H13	119.5
N2—C5—C6	111.7 (3)	C14—C13—H13	119.5
C4—C5—C6	118.8 (3)	C13—C14—C15	119.3 (4)
C1—C6—N1	133.0 (3)	C13—C14—H14	120.4
C1—C6—C5	122.9 (3)	C15—C14—H14	120.4
N1—C6—C5	104.1 (3)	C14—C15—C10	121.2 (4)
N2—C7—N1	115.0 (3)	C14—C15—Br1	118.8 (3)
N2—C7—S1	134.9 (3)	C10—C15—Br1	120.0 (2)
N1—C7—S1	110.1 (3)	C7—N1—C9	115.1 (3)
C9—C8—S1	113.8 (3)	C7—N1—C6	106.0 (3)
C9—C8—H8	123.1	C9—N1—C6	138.8 (3)
S1—C8—H8	123.1	C7—N2—C5	103.1 (3)
C8—C9—N1	110.9 (3)	C7—S1—C8	90.07 (16)
